# Undergraduate nursing students' compatibility with the nursing profession

**DOI:** 10.1186/1472-6920-5-25

**Published:** 2005-07-12

**Authors:** Mohsen Adib-Hajbaghery, Mansur Dianati

**Affiliations:** 1 Faculty of Nursing, Kashan University of Medical Sciences, Kashan, Iran

## Abstract

**Background:**

The high rate of attrition among nursing students has caused some nursing leaders to think about the necessity of considering students' personality during the process of admission into nursing schools. Due to the lack of studies on Iranian nursing students' personality traits, this study was designed to assess freshmen nursing students' personality characteristics and their compatibility with the demands of the nursing profession.

**Methods:**

A descriptive study was conducted at Tehran and kashan medical universities and one of the branches of Azad University. Convenience sampling was used and 52 freshmen nursing students were assessed using Holland's Vocational Interests Inventory.

**Results:**

From the total participants 63.5% were females and 36.5% were males. Based on the Holland's Vocational Interests Inventory 44% did not have appropriate personality characteristics for the nursing profession. 77% of the nursing students participating in the study reported that they lacked information about nursing.

**Conclusion:**

It seems that personality tests can help to select the best students for nursing schools from those who show good academic capabilities. This would decrease the rate of attrition and could improve the quality of care.

## Background

Every profession calls for a special level of knowledge, skills and personal characteristics. If the correspondence between the applicants' individual characteristics and their intended profession is not adequately taken into account, their job compatibility will be hampered [1].

Tests are nowadays used for the selection of people to different professions. In Iran, university entrance exams are administered annually to assess students' suitability for admission into different programs and professions. Administration of such tests is apparently based on a number of assumptions. These assumptions include the following:

- Students' test scores indicate their suitability for the intended program or profession.

- Applicants with higher test score are suitable for more important and sensitive positions.

- Choice of a profession or program is the result of the applicant's informed selection.

- Applicants carefully consider their interests, abilities, and characteristics for selecting an appropriate program or profession.

- Since participation in tests and the choice of program are voluntary, it is assumed that the successful applicants will be highly motivated and will provide high quality services once they get into their profession.

Reports, unfortunately, indicate that this is not always the case. Many nursing students are not well motivated; nurses tend to lose their interest in their jobs early; and despite an increase in the number of nurses with graduate and postgraduate qualifications, the quality of nursing care seems to have declined instead of improving [2,3]. Alikhani (2000) reported that the problem seems to be caused by factors such as wrong selection criteria, teaching methods, content of courses, and methods of evaluation [4]. The purpose of test administration is to select the best candidates for a program or profession. This can be achieved when those who are selected have characteristics compatible with the features of their profession [5].

A review of admission procedures for nursing students in different countries shows that these issues have been largely neglected. Dornik and Vidmar reported that in Slovenia only a high school diploma is required for admission into nursing schools [6]. Similarly, Ehrenfeld et al. as well as the American National League for Nursing argue that admission to nursing schools in America is based on testes similar to the ones for other programs, such as aptitude tests or teacher-made achievement tests [3,7]. Similarly, in Iran, university entrance tests measure only the theoretical knowledge and the aptitude of the applicants; whereas, requirements of the nursing profession are much different and broader. This is why researchers have emphasized, in recent years, that applicants selected for nursing should have the appropriate psychological and personal characteristics in addition to their knowledge and aptitude. To account for this, personality tests have also been recommended to complement educational aptitude tests [7-10].

In his investigations of different professions and job conditions, Holland has determined personality features appropriate for the nursing profession. In his view, nursing requires social personality, even though some degrees of artistic and exploratory personality types may also be desirable [1].

Regarding the importance of correct selection criteria for the nursing profession, the present study was carried out to determine freshman nursing students' personality features and to investigate the compatibility of such features with studying and practicing nursing. It is hoped that the results of the study would help promote nursing services and increase job satisfaction in the profession.

## Methods

This descriptive study was conducted in the nursing schools of Kashan and Tehran medical sciences universities and one of the branches of Azad University (free university), in Iran. The nursing schools of these three universities were selected to represent different types of universities in Iran. These universities admit applicants with varying scores on admission tests and thus the study sample could represent a wider population of students.

The convenience sampling method was used. There were not any exclusion criteria; so, all of the freshmen nursing students in the first semester of the year 2001 were considered as potential samples in each of the three nursing schools (N = 90, 50 from Tehran medical university, 20 from kashan medical university and also 20 from Azad university). After the approval of the schools' principals or the institutional review boards and with the prior consent of the instructors of each group, the potential participants were given an explanation of the study by the researchers during a scheduled session held during the first two month after admission. The questionnaire was introduced and the participants were instructed on how to complete it. Dominant personality features for each personality type-Realistic, Investigative, Artistic, Social, Enterprising, and Conventional – were also explored for the participants. Students who agreed to participate in the study were asked for written consent and then were given the questionnaire to complete in a private place. 75 students volunteered to complete the questionnaire and they were asked to return them within 24 hours.

### Instrument

A two-part questionnaire was used:

**A:) **the first part consisted of a questionnaire on demographic features which also included an item on the participants self-evaluation of their personality type (among the six personality types of: Realistic, Investigative, Artistic, Social, Enterprising, and Conventional), two open-ended questions on the level of familiarity with the nursing profession and their mentality about it at the time of application, and the purpose of choosing nursing as a field of study.

**B): **The second part consisted of Holland's Self-Directed Inventory for Career Development. The Self-Directed Search inventory is one of the most widely used interest inventories [11,12]. It is used widely to assess congruence or "fit" between individuals' personality types and their work or educational environments. The SDS was developed by Holland in 1971 and revised in 1977 and 1985. Internal consistency coefficient on the summary scale of the SDS ranged from 0.90 to 0.94. Test retest reliability correlation of the summery scales also ranged from 0.76 to 0.89 [12-14]. We used the farsi version of SDS that was translated and culturally adapted by Hoseinian and Yazdy, 1995 [1]. This scale consists of two sections. The first, the occupation classification section, includes 500 occupations classified according to six personality styles (i.e.: Realistic, Investigative, Artistic, Social, Enterprising, and Conventional). The second, the Self-directed search (SDS), which comprises of six sub-scales including: occupational daydreams, activities, competencies, occupations, self-estimation, and response organization.

In occupational daydreams, the respondent is asked to name his/her occupational wishes in chronological order. Then a list of 66 activities is presented in the activities subscale.

Activities are presented in 6 sections congruent with the 6 personality types. So, 11 activities stand for each personality type and respondent would express his/her interest as "Like" or "Dislike" for those activities he/she would like or dislike to do.

Subsequently-at the Competencies subscale – respondents respond yes or no to a list of 66 activities that they can or cannot do well or competently. Here also 11 activities stand for each personality type. Then a list of 84 occupations is presented in the occupations sub-scale. These occupations are also presented in 6 sections in accordance with the personality types. So, 14 activities stand for each. The respondent would express his/her interest as "yes" or "no" for those occupations he/she would like or dislike. The fifth subscale is for self-estimation where the respondent would compare him/herself with other persons with the same age. The respondent ranks him/herself on a 1–7 scale in his skills and abilities of different types such as mechanical, scientific, artistic, educational, business, administrative, manual, mathematical, musical, and cooperative skills. This part is presented in two sections each including of 6 types of skills or abilities in accordance with the Holland's personality types.

The final subscale is called the response organization subscale. This is the section for analyzing the participant's responses to the different subscales. Here, scores related to each personality type are calculated. For doing this the "like" and "yes" responses the respondent have given to the subscales of occupation, activities and competencies being calculated for each personality type. The resulted score then will be added up to the person's self scoring in the self-estimation subscale. The possible range of score could be between 0–50 for each personality type. Finally the three personality types with the highest scores will chose to make a three-letter code to indicate the most prominent personality characteristics of person. For instance the following scores could represent a person who has essentially a social character, however she has some considerable degree of investigator and artistic characteristics (i.e. social = 43, investigative = 36, artistic = 29, conventional = 20, enterprising = 20, realistic = 18). So the code SIA will chose to refer to such a person. This not only indicates the most prominent personality characteristics of such individual but also it will be used as a criterion for determining the individual's compatibility with an occupation or a filed of study. In this research we used the personality type with the highest score (i.e. social in the above example) as the most prominent personality character for each student and then we used it as the criterion for determining the student's compatibility with the nursing as a profession or a field of study. So, students with social, artistic and/or investigative characters were considered compatible with the nursing profession.

In this study the participants completed the first questionnaires as well as Holland's Self-Directed Inventory (except for the Professional daydreams and the response organization sections). The researchers analyzed the participants' responses as described above. The collected data were manually analyzed, using descriptive statistics.

Responses to open-ended questions were analyzed through content analysis. These responses were read carefully and their main themes were coded and classified.

## Results

Of 75 questionnaires delivered to the participants, 52 were returned completed. From the total respondents 63.5% were females and 36.5% were males. The mean age of sample was 22 ± 1.2, ranging from 18 to 25 years.

Graph 1 shows the distribution of personality types reported by the participants as well as their personality types according to the Holland's inventory. As the figure shows, 44% of the subjects evaluated themselves as realistic whereas the questionnaire showed only 25% as realistic. The figure also shows that 45% of the participants did not enjoy the personality types appropriate for the nursing profession.

Respondents had been asked how much they knew about nursing at the time of application. About 23% reported average and high levels of information, whereas, 77% knew little about nursing at the time of application. Most of participants did not have a correct picture of the nursing profession and associated it more with medicine. The respondents' presuppositions about nursing fell into four categories: a) Lack of information and no presupposition (30.8%) b) a picture like medicine (40.4%) c) little knowledge (17.3%) d) complete familiarity (11.5%).

For the purpose of entering the program, students' responses fell in the following categories: 1) just entering the university (21.2%), 2) continuing studies in medicine (21.2%), 3) Finding a job (19.2%), 4) lack of attention in choosing a program (15.4%), 5) Service to mankind (13.4%), and 6) Others' influences on course selection (9.6%).

## Discussion

We used Holland's Self-Directed Search (SDS) for assessing Iranian freshmen nursing students' personality characteristics and their compatibility with the demands of the nursing profession. Many studies used SDS for assessing relationship between academic achievement and the students' personality type and confirmed its usefulness for better career planning [15-20]. However, limited publication is available related to using SDS in nursing students.

The present study showed that 45%of the sample lacked the appropriate dominant personality features for nursing. Horn and Holzemer, 1991, were also used SDS questionnaire to compare the personality type of nursing students to women studying engineering. However, they reported that the most of nursing students demonstrated Holland's "social" personality type and engineering students were more "realistic" or "investigative"[21]. However, our finding is similar to the ones reported by Long and Gordon; Fujita et al; Martin et al; and Zolfaghari and Adibi who proposed the use of personality tests for admitting students into nursing programs [10,22-24].

Personality types indicated by the questionnaire did not conform to those found through Holland's self-directed inventory. This maybe partly due to the fact that the subjects were all youngsters who were not personally matured and didn't know their personality well. Marlow has argued that at this stage peoples personality is still being shaped and is, therefore, not stable yet [25].

The results also indicated that most participants did not know the requirements of the profession very well at the time of applying. As Ghazi and Henshaw have claimed, many students enter nursing programs with common sense insights [2]. These people who enter the profession with inadequate knowledge will cease to perform with required standards and experience psychological pressures [5]. Therefore, administrative officials aught to take the necessary steps to help students get adequate information about their planned professions before taking part in the national-wide admission tests. Job councilors believe that successful job selection requires an understanding of individual characteristics, backgrounds, interests and job requirements on the part of both students and selection committees. Otherwise the resulting job-personality incompatibility can lead to poor performance and reduce people's satisfaction and security [5].

The nursing profession and its requirements call for persons with social, artistic and investigative personality types and with characteristics such as patience, tolerance, friendliness, love and sense of cooperation and responsibility [21]. These characteristics are not usually tested in current selection tests for the nursing profession. Ghazi and Henshaw as well as Ehrenfeld et al. have also reported this fact and have attributed to it such factors as lack of motivation during the study period, low achievement, high attrition rates, lack of interest in the job and quitting the job [2,7].

The purpose of the nation-wide university entrance exam in Iran is to evaluate the applicants' readiness for different educational programs and their corresponding professions and to select the best candidates for them. Yet, this annual exam has a fixed format for all disciplines and dose not include any components on the evaluation of the applicants' psychological traits and required personality features. Based on the findings of this study, it is recommended that personality and interest tests be used to supplement the existing academic and aptitude tests of admission into nursing programs. In other words applicants can be short-listed based on their academic capabilities. Once twice the number of eligible applicants for each nursing school has been short listed, interest and personality tests can be used to select the best applicants. This can greatly reduce students' attrition rates. It can also increase job satisfaction and would guarantee the quality of nursing care provided by the future nurses.

This was the first step in the investigation of job-personality compatibility among a limited sample of Iranian nursing students. So the results may not be necessarily be generalizable to all nursing students. The replication of this study on a larger sample is recommended.

## Conclusion

The present study showed that many nursing students lacked the appropriate personality features for nursing. The results also indicated that most participants did not know the requirements of the profession at the time of applying. Therefore, administrative officials aught to take the necessary steps to help students get adequate information related to their planned professions before taking part in admission test. It seems that personality tests can help to select the best students for nursing schools from those who show good academic capabilities. This would decrease the rate of attrition and could improve the quality of care.

## List of abbreviations used

Self-Directed Search (SDS).

## Competing interests

The author(s) declare that they have no competing interests.

## Authors' contributions

MAH: Initiation and design of the research, collection and analysis of the data and writing the paper.

MD: helping in data gathering and revision of draft paper.

**Figure 1:**
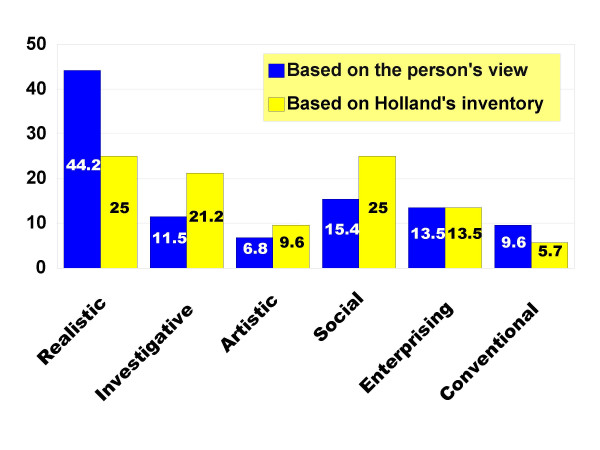
The distribution of personality types reported by the participants as well as their personality types according to the Holland's inventory.

## Pre-publication history

The pre-publication history for this paper can be accessed here:


